# Molecular characterization of *Cytauxzoon brasiliensis* in domestic cats (*Felis catus*) from Rio de Janeiro state, Brazil: hematological findings

**DOI:** 10.1007/s11259-026-11385-z

**Published:** 2026-07-07

**Authors:** Ellen Meireles Brandão, Jônathan David Ribas Chagas, Isadora dos Santos Dias, Thaís Silva Oliveira, Gilliard do Nascimento Ferreira, Aroldo de Souza Junior, Huarrisson Azevedo Santos, Matheus Dias Cordeiro, Cláudia Bezerra da Silva, Bruna de Azevedo Baêta

**Affiliations:** 1 Department of Animal Parasitology, Veterinary Institute, Federal Rural University of Rio de Janeiro, BR465-km07, Seropédica, Rio de Janeiro, 23890-000 Brazil; 2 Department of Epidemiology and Public Health, Veterinary Institute, Federal Rural University of Rio de Janeiro, BR465-km07, Seropédica, Rio de Janeiro, 23890-000 Brazil

**Keywords:** *Cytauxzoon brasiliensis*, Domestic cats, Molecular characterization, Piroplasmids, Hematology

## Abstract

*Cytauxzoon* spp. are tick-borne haemoprotozoans that infect domestic and wild felids and are of increasing relevance in veterinary medicine and epidemiology of vector-borne pathogens. In Brazil, the genetic diversity of these agents in wild felids has been progressively expanded, culminating in the description of *Cytauxzoon brasiliensis*. However, the occurrence of this species in domestic cats is unknown. Therefore, the present study aimed to detect and molecularly characterize *C. brasiliensis* in domestic cats from the state of Rio de Janeiro, Brazil, and to describe the main hematological findings observed in positive animals. A total of 404 blood samples from domestic cats were analyzed using nested PCR targeting the 18S rRNA gene, followed by molecular characterization using the mitochondrial *cytB* gene. Positive samples were subjected to similarity, phylogenetic, and haplotype diversity analyses. Hematological data from positive animals were also evaluated, along with differential molecular diagnoses for other hemoparasites and infectious agents. Four samples (~ 1%) were positive for *Cytauxzoon* sp. in the 18S rRNA-based assay, and three showed intraerythrocytic inclusions suggestive of piroplasms in blood smears. The sequences obtained clustered phylogenetically with isolates previously identified as *C. brasiliensis* in Brazilian wild felids, confirming the presence of this agent in domestic cats. Hematological analyses revealed thrombocytopenia in all four positive animals, anemia in two animals, and variable leukocyte abnormalities. No molecular detection of *Bartonella* spp., hemoplasmas, members of the family Anaplasmataceae, or *Leishmania infantum chagasi*, was observed. These results demonstrate, for the first time, the infection of domestic cats by *C. brasiliensis* in the state of Rio de Janeiro and reinforce the need to include this agent in the differential diagnosis of feline hemoparasitoses. In addition, the findings suggest that infection may be associated with relevant hematological alterations, although studies including a larger number of animals and a more robust clinical-epidemiological design are still needed to clarify its actual pathogenic impact on the host.

**Supplementary Information:**

The online version contains supplementary material available at https://doi.org/10.1007/s11259-026-11385-z.

## Introduction

The genus *Cytauxzoon* (Apicomplexa: Theileriidae) comprises tick-borne hemoparasites that infect wild and domestic felids in different regions of the world. Interest in these agents initially increased following the description of feline cytauxzoonosis in the United States, where *Cytauxzoon felis* is recognized as the causative agent of an acute, systemic, and frequently fatal disease in domestic cats. However, with the increased use of more sensitive molecular methods, it has become evident that *Cytauxzoon* infection in felids has a broader spectrum, including subclinical infections and chronic carrier states, particularly in certain geographical regions and epidemiological contexts (de Oliveira et al. 2022).

In recent years, knowledge of the genus *Cytauxzoon* has expanded substantially, beyond North America. In Europe, new species have been described in wild felids (Panait et al. 2021), whereas in Asia, *Cytauxzoon* infections have been reported in both domestic felids, revealing a considerable genetic diversity within the genus (Zou et al. 2019; Rahmati Moghaddam et al. 2020). In South America, studies have shown that the genetic diversity of this group is greater than that initially assumed. In Brazil, the first reports in domestic cats demonstrated the occurrence of *Cytauxzoon* spp. in clinically healthy animals, suggesting that the infection may silently circulate in this host. However, most of these studies were based on short fragments of the 18S rRNA gene, a marker that is useful for screening but has limited resolution for discriminating between phylogenetically closely related species. Thus, although these studies were fundamental in demonstrating the circulation of the parasite in domestic cats, the actual taxonomic identity of the isolates detected in Brazil remains uncertain (Raimundo et al. 2021; André et al. 2022).

This limitation became even more relevant after the recent taxonomic revision of the group in South America. In 2024, *Cytauxzoon brasiliensis* was formally described in *Leopardus tigrinus* based on morphological, histopathological, and molecular evidence, including nuclear and mitochondrial markers (Duarte et al. 2024). Subsequently, broader phylogenetic analyses have reinforced that the isolated use of short 18S rRNA fragments is insufficient for the reliable delimitation of species within the genus, whereas mitochondrial markers, such as *cytB*, provide greater discriminatory power and increased robustness for taxonomic inferences (Calchi et al. 2025). Thus, the description of *C. brasiliensis* required a cautious reinterpretation of some previous reports of *Cytauxzoon* sp. in domestic cats in Brazil, since it remained unclear whether these animals could act as hosts for this newly described species.

In parallel with this taxonomic uncertainty, important epidemiological and clinical laboratory gaps persist. In South America, the *Cytauxzoon* spp. vector has not yet been definitively demonstrated. *Amblyomma sculptum* has been proposed as a putative vector based on epidemiological and molecular evidence; however, its vector competence remains unconfirmed experimentally (Fagundes-Moreira et al. 2022; Calchi et al. 2025). In addition, although recent studies have indicated that *Cytauxzoon* infection in felids may range from apparently subclinical forms to severe clinical disease, depending on the species involved, the host, and the presence of co-infections, information on the potential hematological impact of *C. brasiliensis* infection in domestic cats remains scarce (de Oliveira et al. 2022; Fenelon et al. 2023; Reichard et al. 2024).

Therefore, at least three central questions remain unanswered: whether *C. brasiliensis* occurs in domestic cats, whether molecular markers with greater resolving power would confirm its circulation in this host, and whether infection would be accompanied by detectable hematological alterations. In this context, the present study aimed to detect and molecularly characterize *C. brasiliensis* in domestic cats (*Felis catus*) from the state of Rio de Janeiro, Brazil, and to describe the hematological findings observed in positive animals.

## Material and methods

### Study site and blood sample collection

The study was conducted between September 2024 and January 2026 and included 404 residual blood samples from domestic cats submitted for routine diagnostic testing and provided by a veterinary clinical laboratory in Rio de Janeiro, Brazil. This was a convenience sampling approach with no predefined inclusion criteria regarding sex, age, or breed. The samples originated from two mesoregions of the state of Rio de Janeiro: the Metropolitan region (*n* = 198) and the South Fluminense region (*n* = 206). The Metropolitan mesoregion comprises 30 municipalities, covering an area of 10,263 km², with a mean altitude of 177 m and a tropical climate. The South Fluminense mesoregion comprises 14 municipalities, covering an area of 7,966 km², with a mean altitude of 357 m and a predominantly subtropical climate.

### Hematological analysis

The complete blood counts corresponding to the samples included in the study were provided by the source laboratory, allowing for the complementary evaluation of the positive animals. Hematological analysis was performed using an automated hematology analyzer (URIT 5160 VET; MHLAB^®^), which provided the following parameters: total leukocyte count (WBC), leukocyte differential count, erythrocyte count (RBC), hemoglobin (HGB), hematocrit (HCT), mean corpuscular volume (MCH), mean corpuscular hemoglobin concentration (MCHC), and platelet count (PLT). Blood smears were prepared, stained with Panótico Rápido^®^ (a rapid Romanowsky-type stain), and examined under a light microscope (1000×) (Laborcare L3000T PI^®^) for morphological verification of the automated results and screening for the presence of hemoparasites.

For samples in which intraerythrocytic inclusions suggestive of piroplasms of the genus *Cytauxzoon* were observed, new blood smears were prepared and stained with Giemsa (1:10) to allow for better morphological characterization of the parasitic structures.

The degree of parasitemia was estimated by counting the number of infected erythrocytes among 2000 erythrocytes using an Olympus BX51 microscope at ×1000 magnification. Morphometric analysis of well-defined organisms with characteristic morphologies (*n* = 100) (Panait et al. 2020) was performed using a Zeiss Axio Cam HRc camera and CellSens software.

### DNA extraction and endogenous gene verification

DNA was extracted from the samples using the commercial Wizard Genomic DNA Purification Kit (Promega^®^) according to the manufacturer’s recommended protocol. After purification, the DNA was stored at − 20 °C until molecular analysis was performed. To verify the suitability of the DNA for molecular analyses, all samples were initially subjected to partial amplification of the endogenous *GAPDH* gene, which encodes glyceraldehyde-3-phosphate dehydrogenase, generating a 400-bp fragment (Birkenheuer et al. 2003). Amplification of this target was used as an endogenous quality control for extracted DNA.

### Molecular detection of piroplasmids based on the 18S rRNA gene and molecular characterization of *Cytauxzoon* spp. based on the *cytB* gene

The samples were initially subjected to nested PCR to amplify a fragment of the 18 S rRNA gene (~ 800 bp) to screen for piroplasmids of the order Piroplasmida (Jefferies et al. 2007). The samples were subsequently subjected to amplification of a fragment of the mitochondrial *cytB* gene (~ 1444 bp), which was used for the molecular characterization of *Cytauxzoon* spp. (Schreeg et al. 2013; Panait et al. 2021). DNA from *Babesia vogeli* obtained from a naturally infected dog was used as a positive control for the 18 S rRNA assay, whereas DNA from *C. felis* obtained from a naturally infected domestic cat (*F. catus*) from Seropédica, Rio de Janeiro State, Brazil, previously confirmed by DNA sequencing, was used as a positive control for the *cytB* assay. Ultrapure water was used as a negative control for all reactions. The details of the primers and amplification conditions are described in Supplementary Material 1.

### Differential diagnosis for other hemoparasites

Molecular analyses of *Cytauxzoon* sp. positive samples for differential diagnosis included the detection of *Bartonella* spp. through amplification of the *ITS* gene (400–600 bp), using previously tested DNA from *Bartonella henselae* as a positive control. For hemoplasmas and other members of the class Mollicutes, amplification of the 16S rRNA gene (270 bp) was performed, using DNA from *Mycoplasma haemofelis* obtained from naturally infected cats as the positive control. For members of the family Anaplasmataceae, screening was based on PCR targeting the 16S rRNA gene (345 bp), using DNA from *Anaplasma marginale* strain AmRio2 and *Ehrlichia canis* as the positive controls. For *Leishmania infantum chagasi*, amplification of the 18S rRNA gene (358 bp) was performed using DNA from a naturally infected dog as the positive control. Ultrapure water was used as a negative control in all assays. The details of the protocols are described in Supplementary Material 1.

### Separation of PCR products by electrophoresis

PCR products were subjected to electrophoresis on a 1.5% agarose gel (UltraPure™ LMP Agarose, Invitrogen^®^) at 75 V (5 V/cm) for 45 min in TAE buffer (Tris-acetate-EDTA), stained with ethidium bromide (0.5 µg/mL), and visualized using an ultraviolet light transilluminator (L-PIX Sti, Loccus). The sizes of the amplified fragments were estimated by comparison with a 100 bp molecular weight marker (100 bp Ladder Plus, Ready-To-Use, M1071, Sinapse Inc^®^).

### PCR purification and sequencing

For sequencing, 5 µL of PCR products obtained from samples positive for the 18S rRNA and *cytB* genes were subjected to enzymatic purification with ExoSAP-IT™ (Thermo Fisher Scientific^®^), according to the manufacturer’s recommendations, to remove residual primers and deoxynucleoside triphosphates (dNTPs). The purified amplicons were sequenced by the Sanger method in both directions using the respective primers employed in the amplification reactions on an ABI 3500 Genetic Analyzer (Applied Biosystems^®^).

### Haplotype analysis

The genetic diversity of *C. brasiliensis* was evaluated in an exploratory manner based on the sequences obtained in this study and the homologous sequences available from wild hosts. Polymorphism indices were estimated using DnaSP v6.12.03, including the number of polymorphic sites (S), number of haplotypes (H), haplotype diversity (Hd), and nucleotide diversity (π) (Rozas et al. 2017). Estimates were calculated from the final alignment, excluding only ambiguous positions/gaps to preserve the contribution of invariant sites to the calculation of nucleotide diversity. The haplotype distribution was visualized using a median-joining network in PopART v1.7. The analyzed dataset included sequences generated in this study and previously published sequences from Brazilian wild felids.

### Phylogenetic analysis

Chromatograms were manually inspected, and nucleotide sequences were assembled and edited using the CLC Main Workbench 23 software (CLC Bio-Qiagen). The consensus sequences were then subjected to similarity analysis using BLASTn in the GenBank/NCBI database. Previously published homologous sequences were retrieved in FASTA format and aligned with the sequences obtained in this study using the ClustalW algorithm implemented in MEGA X software (Kumar et al. 2018).

Phylogenetic analysis of *C. brasiliensis* was performed based on datasets containing 775 bp of the 18S rRNA gene and 981 bp of the *cytB* gene after alignment and trimming to comparable homologous regions. The 18S rRNA dataset included 30 *Cytauxzoon* spp. sequences, whereas the *cytB* dataset included 39 sequences. The outgroup sequences used were *Babesia bovis* (L19077) for 18S rRNA and *Hepatozoon canis* (MK214285) for the *cytB*.

Phylogenetic trees were inferred using the Maximum Likelihood (ML) method, applying the Kimura 2-parameter (K2) substitution model for the 18S rRNA gene and the Hasegawa-Kishino-Yano model with gamma distribution and invariant sites (HKY + G+I) for the *cytB* gene. Analyses were performed using MEGA X (Kumar et al. 2018), with evolutionary model selection based on the Akaike Information Criterion (AIC). Statistical support for the clades was estimated using bootstrap analysis with 1,000 replicates using a heuristic search. The final editing of the trees was performed using Inkscape v1.3.2.

## Results

**Fig. 1:**
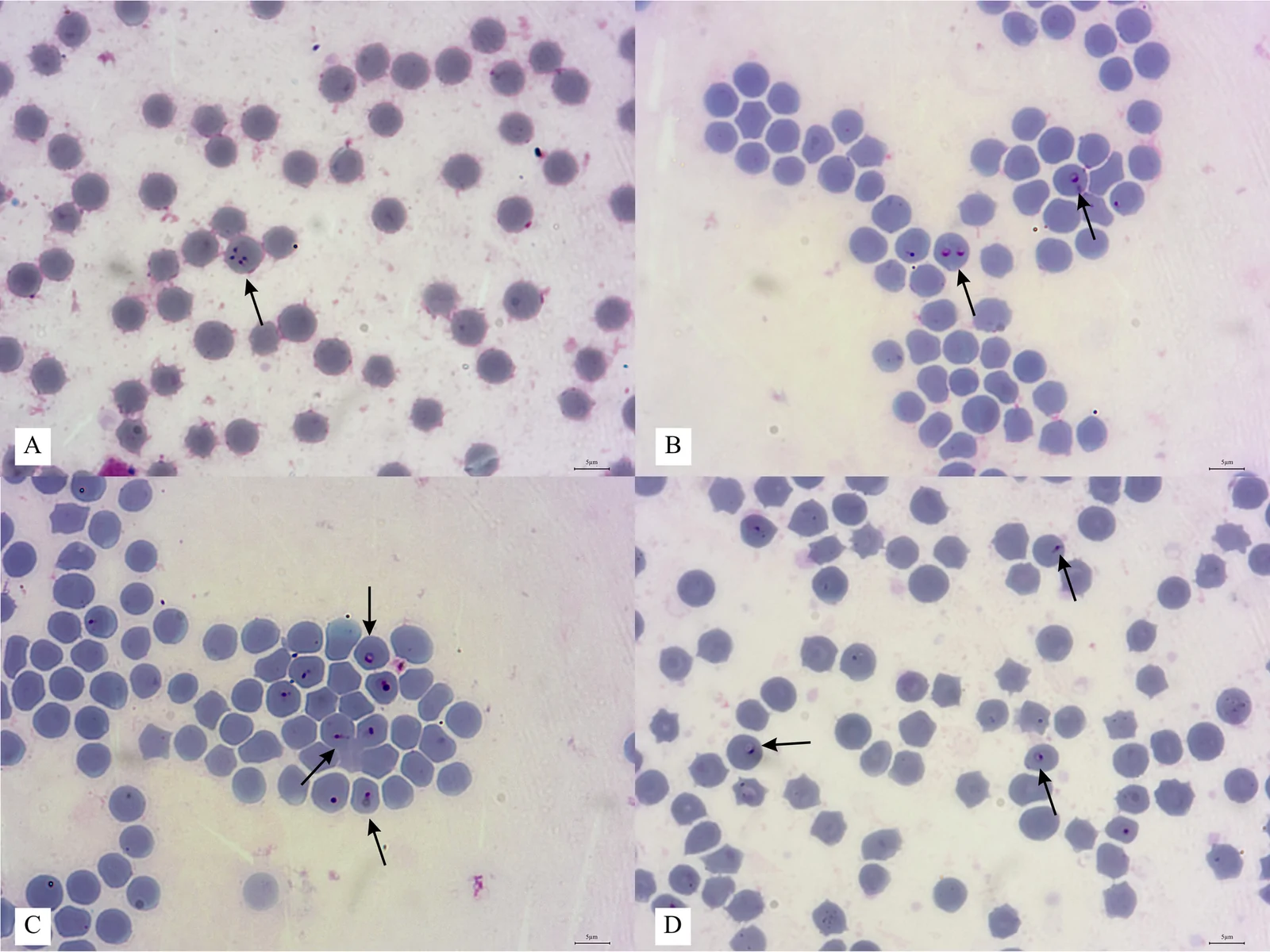
(**A**) Intraerythrocytic tetrad-shaped inclusions, indicated by arrows. (**B**) Signet-ring-shaped inclusions, morphologically compatible with *Cytauxzoon* spp. (**C**–**D**) Ring-shaped and comma-shaped intraerythrocytic inclusions, indicated by arrows, observed in stained blood smears from domestic cats (*Felis catus*) in Rio de Janeiro, Brazil. Images were obtained using light microscopy with an oil-immersion objective at 1000× magnification. Scale bar = 5 μm

In the evaluation of blood smears, three animals showed intraerythrocytic inclusions morphologically compatible with piroplasmids, including round to oval signet ring–shaped organisms, comma-shaped forms within erythrocytes and tetrad-shaped inclusions (Maltese cross) were also observed (Fig. 1).

The mean length of the well-defined forms was 3.0 ± 0.7 μm, while the mean width was 2.0 ± 0.6 μm in cat 2. In cat 3, the mean length was 2.0 ± 0.5 μm and the mean width was 2.0 ± 0.4 μm. The parasitemia levels were estimated at 0.75% in cat 2 and 2.05% in cat 3.

All samples showed amplification of the endogenous *gapdh* gene, indicating adequate DNA quality and successful amplification. In molecular screening based on the 18S rRNA gene, four samples (~ 1%; 4/404) tested positive for piroplasmids compatible with *Cytauxzoon* sp., including the three that were positive on blood smear examination (Supplementary Material 1). All positive cats were male and mixed-breed; three were 2 years old and one was 3 years old. These samples were used for subsequent molecular characterization analyses.

In the BLASTn analysis, the 18S rRNA gene sequences obtained from domestic cats showed 100% identity with *C. brasiliensis* sequences previously detected in *L. tigrinus* and *Leopardus pardalis* from Brazil (PP583821, PQ686812, PQ686811, and PQ724418). For the *cytB* gene, the sequences exhibited 97–99% identity with *C. brasiliensis* isolates detected in *Puma concolor*, *L. tigrinus*, and *L. pardalis* in Brazil (PQ667868; PP588457; PQ724035; PQ667867), corroborating the taxonomic placement of the samples (18S rRNA: PZ364081-PZ364084; *cytB*: PZ371847-PZ371850).

**Fig. 2:**
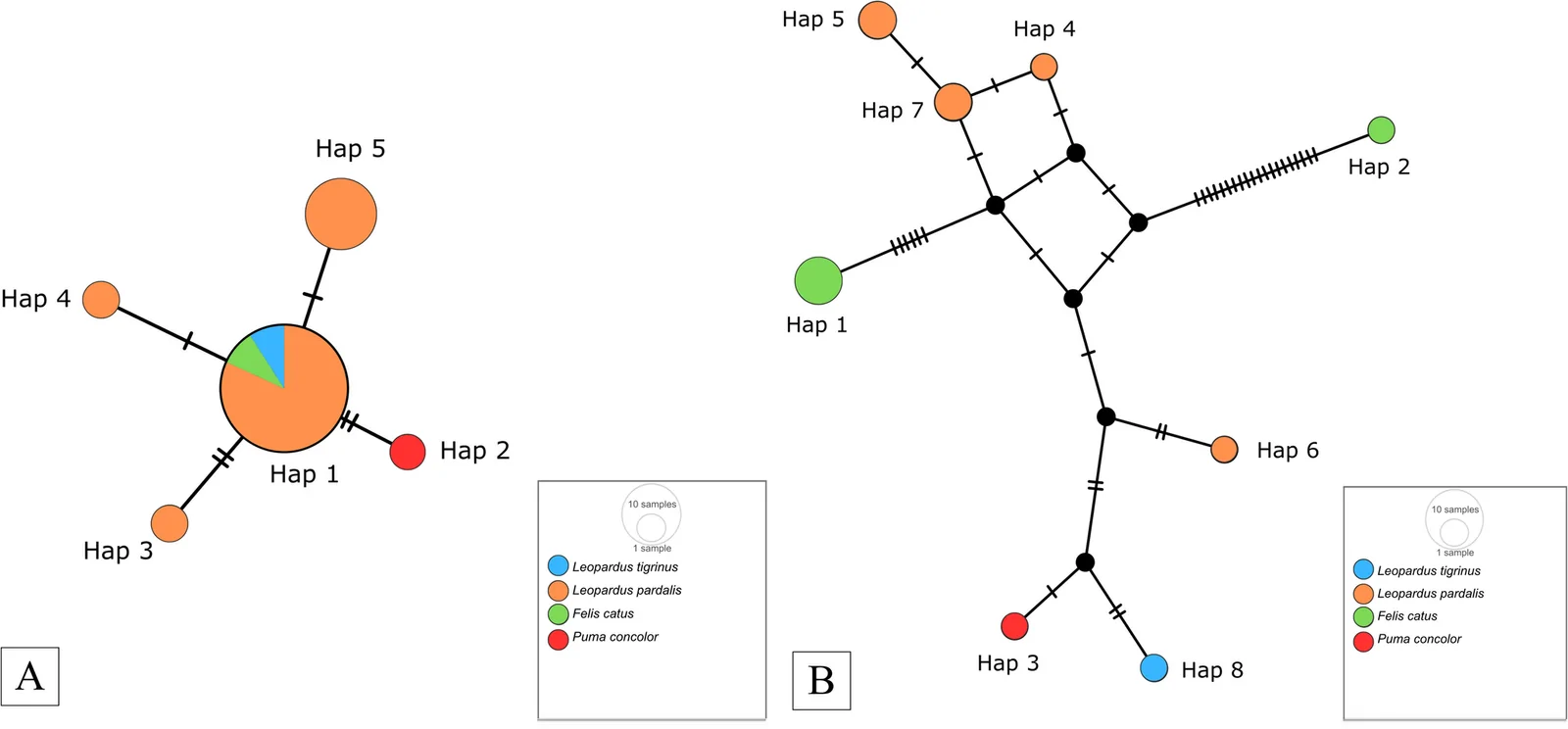
Median-joining haplotype network based on 18S rRNA (**A**) and *cytB* (**B**) sequences of *Cytauxzoon brasiliensis* isolates detected in domestic cats and wild felids in Brazil. Small marks between the haplotypes represent mutational steps. Black circles indicate inferred intermediate haplotypes based on single nucleotide polymorphisms (SNPs)

Haplotype analysis revealed lower variability among the 18S rRNA sequences and greater discriminatory power for the *cytB* gene. In the 18S rRNA dataset, five haplotypes were identified, with Hap 1 representing the samples used in this study. For *cytB*, a higher number of haplotypes and greater resolution for differentiating isolates were observed, reinforcing the usefulness of this marker for the intraspecific characterization of *C. brasiliensis* (Fig. 2).

**Fig. 3:**
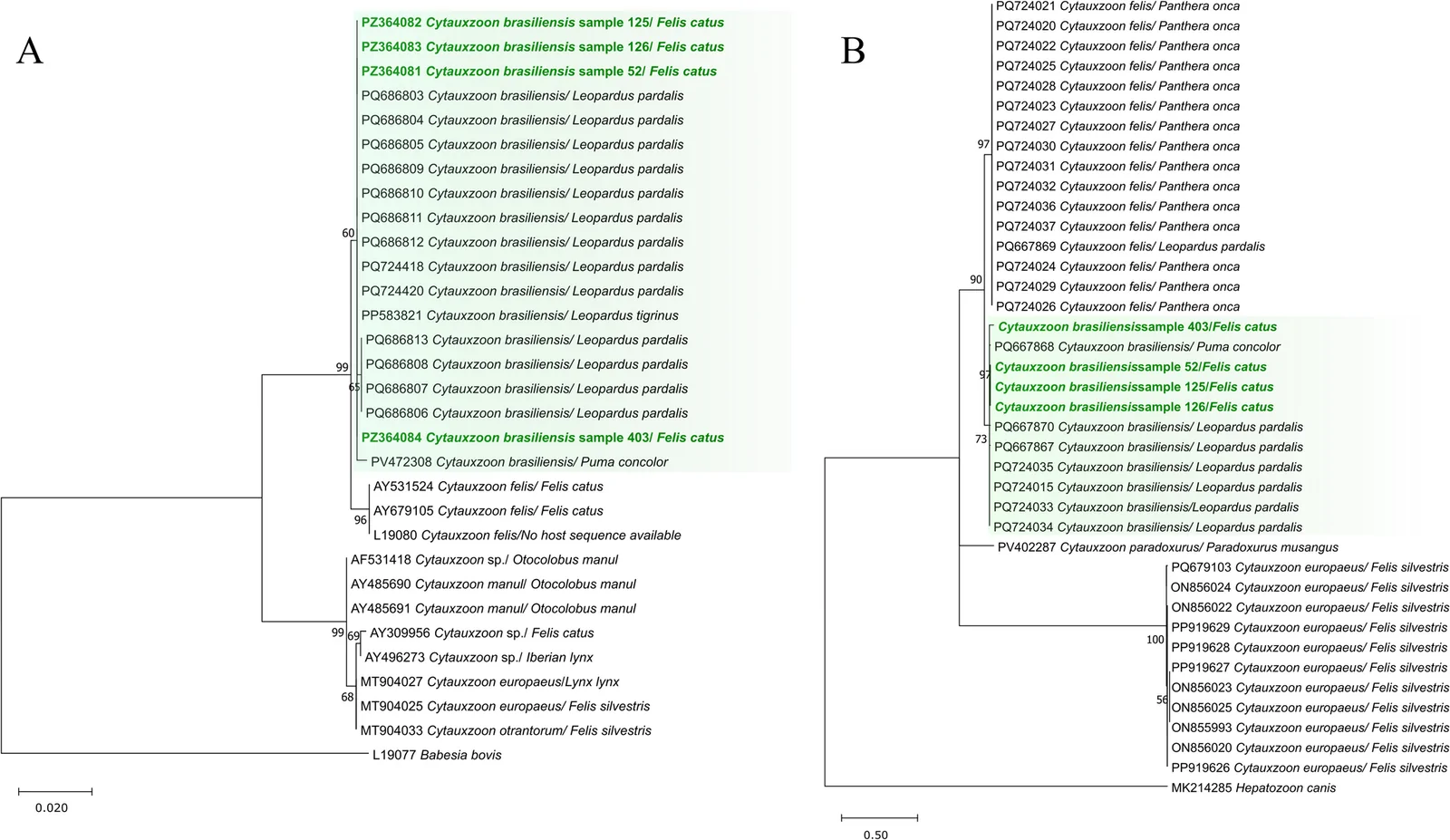
(**A**) Maximum likelihood phylogenetic tree based on a 775 bp fragment of the 18S rRNA gene of *Cytauxzoon* spp., inferred under the Kimura 2-parameter substitution model. The analysis included 31 sequences in total. (**B**) Maximum likelihood phylogenetic tree based on a 981 bp fragment of the *cytB* gene of *Cytauxzoon* spp., inferred under the Hasegawa-Kishino-Yano substitution model. Bootstrap values are indicated next to the corresponding branches. Differences in the evolutionary rates among sites were modeled using a discrete gamma distribution (G + I). The scale bar represents the number of nucleotide substitutions per site. The analysis included 40 sequences in total

Phylogenetic analysis based on the 18S rRNA gene showed that the sequences generated in this study clustered within the *C. brasiliensis* clade, together with isolates previously reported in Brazilian wild felids (Fig. 3A). Although this marker was suitable for confirming the taxonomic affinity of the samples with *C. brasiliensis*, it showed limited resolution in distinguishing finer phylogenetic relationships among isolates.

Phylogenetic analysis based on the *cytB* gene showed that the sequences generated in this study clustered with previously described *C. brasiliensis* isolates with high statistical support (Fig. 3B). This analysis also revealed a consistent phylogenetic separation between *C. brasiliensis* and other *Cytauxzoon* species, highlighting the usefulness of this mitochondrial marker for taxonomic inferences. Furthermore, the clustering pattern suggests the circulation of closely related *C. brasiliensis* lineages among wild and domestic Brazilian felids.

The hematological values of the four positive cats are listed in Table 1. Thrombocytopenia was observed in all animals, and anemia normocytic, normochromic, and non-regenerative was observed in two animals. Cats 2 and 3 showed leukocytosis with neutrophilia and a left shift, whereas cat 1 exhibited leukopenia and neutropenia. Lymphopenia was observed in all four cats in this study. Additionally, cat 1 presented toxic changes in neutrophils, characterized by cytoplasmic basophilia and Döhle bodies, whereas cat 3 exhibited toxic changes in neutrophils, including cytoplasmic basophilia and neutrophils with ring-shaped nuclei, along with marked icterus. Given the limited number of cases, these findings should be interpreted descriptively rather than inferentially.

**Table 1:** Hematological parameters of domestic cats (*F. catus*) naturally infected with *C. brasiliensis* in the state of Rio de Janeiro, Brazil

Parameters	Cat 1 (ID 52)	Cat 2 (ID 125)	Cat 3 (ID 126)	Cat 4 (ID 403)	Reference values*
Erythrocytes (×10⁶/µL)	8.29	3.51	4.67	7.32	5.0–10.0
Hemoglobin (g/dL)	12.5	5.7	7.1	9.6	8–15
Hematocrit (%)	37	16	21	30	24–45
MCH (fL)**	44.63	45.58	44.97	40.98	39–55
MCHC (%)**	33.7	35.6	33.8	32.0	30–36
Total leukocyte count (/µL)	1.400	17.300	29.000	11.200	5.500–19.500
Basophils (/µL)	0	0	0	0	Rare
Eosinophils (/µL)	0	0	0	112	0–1.500
Band neutrophils (/µL)	140	519	1,160	0	0–300
Segmented neutrophils (/µL)	1.148	16.089	26.970	9.968	2.500–12.500
Lymphocytes (/µL)	98	692	870	1,120	1.500–7.000
Monocytes (/µL)	14	0	0	0	0–850
Platelets (/µL)	150.000	71.000	49.000	186.000	230.000–680.000

*Reference: *Schalm’s Veterinary Hematology* (2010)

**MCH mean corpuscular volume, *MCHC *mean corpuscular hemoglobin concentration

In the molecular assays performed for differential diagnosis, *Bartonella* spp., agents of the class Mollicutes, members of the family Anaplasmataceae, and *Leishmania infantum chagasi* were not detected in the samples positive for *C. brasiliensis*.

## Discussion

The molecular detection of *C. brasiliensis* in domestic cats (*Felis catus*) in the state of Rio de Janeiro expands the current knowledge of the epidemiology of this piroplasmid in Brazil. To date, *C. brasiliensis* has been formally described in Brazilian wild felids, and its identification in domestic cats suggests that genetically related lineages circulate among different felid hosts (Duarte et al. 2024; Calchi et al. 2025). This finding is particularly relevant because some previous reports on Brazilian domestic cats were based on short fragments of the 18S rRNA gene, which have limited discriminatory power for closely related *Cytauxzoon* species.

The detection frequency observed in this study (~ 1%) was low and, in general, consistent with previous reports of *Cytauxzoon* spp. in domestic cats in Brazil, particularly in studies based on convenience sampling and animals from urban or periurban environments (Raimundo et al. 2021; André et al. 2022). However, the epidemiological interpretation of this percentage should be made with caution, as the retrospective design, lack of standardized clinical information, and laboratory-based origin of the samples limit inferences regarding the prevalence, risk factors, and spatial distribution of infection.

Microscopic examination of blood smears revealed parasitaemia levels of 0.75% and 2.05% in the studied cats. These findings are consistent with previous reports describing low parasitaemia in felines infected with *Cytauxzoon* spp. (Carli et al. 2014; Legroux et al. 2017). In contrast, Panait et al. (2020) reported a parasitaemia level of 33%, highlighting the possible variability in parasite burden among infected felines.

The hematological findings observed in the positive cats warrant attention, particularly because of the consistent presence of thrombocytopenia and the occurrence of anemia in two animals. However, thrombocytopenia is a common and often nonspecific hematological abnormality in cats and may be associated with a wide range of conditions, including inflammatory and infectious diseases, retroviral infections, neoplasia, immune-mediated disorders, and physiological responses such as stress (LeVine et al. 2024). Importantly, pseudothrombocytopenia due to platelet aggregation was ruled out by careful evaluation of blood smears. In addition, the limited number of cases and the absence of a systematic investigation of other relevant feline conditions preclude attributing direct causality to *C. brasiliensis* infection. Therefore, these results should be interpreted as descriptive evidence of possible clinical and laboratory repercussions rather than conclusive evidence of pathogenicity.

From a clinical perspective, the hematological profile described in the positive animals partially resembles the alterations reported in cases of cytauxzoonosis caused by *C. felis*, in which anemia, thrombocytopenia, and leukocyte abnormalities, such as neutrophilic leukocytosis and left shift, are frequently observed, particularly in acute disease (Schreeg et al. 2013; Reichard et al. 2024). The anemia observed in the present study was characterized as normocytic, normochromic, and non-regenerative in the two affected cats. In particular, Rahmati Moghaddam et al. (2020) reported similar findings in naturally infected domestic cats, including thrombocytopenia, anemia, neutrophilic leukocytosis, hyperproteinemia, and hyperbilirubinemia. Similarly, in the present study, thrombocytopenia was observed in all four *C. brasiliensis*-positive cats, anemia was detected in two animals, and cats 2 and 3 showed leukocytosis with neutrophilia and a left shift. In addition, marked icterus was observed in cat 3, a finding that may be compatible with the hyperbilirubinemia described in previous cases of feline cytauxzoonosis. However, serum bilirubin and total plasma protein were not systematically evaluated in the present study, which limits a direct comparison with the biochemical abnormalities reported by Rahmati Moghaddam et al. (2020). Moreover, because previous comparative studies mainly involved *C. felis*, whereas the present study detected *C. brasiliensis*, these similarities should be interpreted cautiously and should not be used to infer equivalent pathogenicity between the two species.

The pathophysiological mechanisms underlying these alterations are likely multifactorial. Anemia in cytauxzoonosis has been associated with both hemolytic processes and inflammatory-mediated suppression of erythropoiesis, with variable regenerative responses depending on disease stage and survival. Leukocytosis with neutrophilia and left shift is consistent with a systemic inflammatory response driven by cytokine release, whereas in severe or terminal cases, bone marrow infiltration and dysfunction may result in pancytopenia (Rahmati Moghaddam et al. 2020). Thrombocytopenia has traditionally been attributed to disseminated intravascular coagulation (DIC), a recognized complication of feline cytauxzoonosis (Conner et al. 2015), although platelet consumption secondary to systemic inflammation and endothelial activation may also contribute.

Despite these similarities with previously reported patterns in *C. felis* infection, the absence of direct assessment of tissue schizogony in this study and the limited sample size preclude extrapolation of disease severity or pathogenic mechanisms. Thus, while the observed hematological changes are consistent with those described in feline cytauxzoonosis, they cannot be used to equate the clinical impact of *C. brasiliensis* with that of *C. felis* in North America.

The absence of molecular detection of *Bartonella* spp., hemoplasmas, Anaplasmataceae, and *Leishmania infantum chagasi* reduces the likelihood that these agents contributed to the hematological changes observed in the *C. brasiliensis*-positive cats. However, additional coinfections and comorbidities not assessed in the present study may have acted as confounding factors. In particular, feline retroviral infections, such as feline immunodeficiency virus (FIV) and feline leukemia virus (FeLV), were not investigated. These infections are important differential diagnoses in domestic cats and may be associated with hematological abnormalities similar to those observed here, including anemia, thrombocytopenia, leukopenia, lymphopenia, and neutropenia. Therefore, their potential contribution to the hematological findings cannot be excluded, and the alterations observed in the positive cats should be interpreted descriptively rather than as evidence of a direct causal association with *C. brasiliensis* infection. This caveat is important, as cats with concomitant detection of *Cytauxzoon* sp. and other infectious agents, including *Mycoplasma haemofelis* and FIV, have already been reported in Brazil, which may modify hematological and clinical presentation (Fenelon et al. 2023). Future prospective studies including systematic FeLV/FIV testing, clinical follow-up, and broader investigation of comorbidities are needed to clarify the pathogenic role of *C. brasiliensis* in domestic cats.

From an epidemiological standpoint, the identification of *C. brasiliensis* in domestic cats is consistent with the hypothesis of lineage sharing between wild and domestic felids in interface areas between natural and anthropized environments. Similar patterns of interspecific circulation have been suggested in other regions, including Russia (Naidenko et al. 2022). However, the data from this study alone do not allow confirmation of spillover events, as such an inference would require integrated sampling of domestic hosts, wildlife, and vectors, as well as more detailed ecological and spatial information. This finding reinforces the need for surveillance in regions with forest remnants and circulation of wild felids.

Although the vector of *Cytauxzoon* spp. in Brazil remains undefined, *Amblyomma sculptum* has been proposed as a putative vector based on its epidemiological compatibility with areas where the parasite occurs; however, its vector competence has not yet been experimentally demonstrated (Fagundes-Moreira et al. 2022; Calchi et al. 2025). Therefore, any inferences regarding transmission in the present study should be made with caution. Investigations involving ticks collected from domestic cats and wild felids, combined with molecular and experimental approaches, are essential to clarify the *C. brasiliensis* transmission cycle.

The molecular and phylogenetic analyses performed in this study reinforce the importance of using mitochondrial markers, especially the *cytB* gene, for the taxonomic characterization of *Cytauxzoon* sp. in the South American context. The 100% identity observed for 18S rRNA confirms the affinity of the samples with *C. brasiliensis* but also highlights the limitation of this marker for fine discrimination among closely related isolates. In contrast, the better performance of the *cytB* gene in haplotype and phylogenetic analyses supports the recent recommendation that mitochondrial genes should be prioritized in studies of diversity and species delimitation within the genus (Duarte et al. 2024; Calchi et al. 2025).

The phylogenetic analysis and the context of previous reports suggest that more than one *Cytauxzoon* lineage/species may be circulating in different geographic regions, including South America. In the present study, *C. brasiliensis* was identified in domestic cats in Brazil, while previous molecular evidence has also indicated the occurrence of other *Cytauxzoon* lineages in wild felids (Calchi et al. 2025). Together, these findings support the possibility of a broader diversity within the genus circulating in the country.

Furthermore, the haplotype analysis performed in the present study should be interpreted with caution, as it was based on only four positive domestic cat samples and a limited number of homologous sequences available from GenBank. Therefore, the haplotype network should be considered an exploratory visualization of preliminary genetic relationships rather than evidence of population structure, geographic clustering, or the full genetic diversity of *C. brasiliensis*. Future studies including larger numbers of positive samples, broader geographic coverage, and additional sequences from domestic and wild felids are needed to validate the genetic patterns observed herein.

Taken together, these results demonstrate that *C. brasiliensis* can be detected in domestic cats from the state of Rio de Janeiro and that relevant hematological alterations occurred in the positive animals evaluated in this study. However, the magnitude of the pathogenic role of this agent in *F. catus* remains to be defined through prospective studies with larger sample sizes, clinical follow-up, investigation of co-infections, and assessment of potential vectors. Such data will be essential to establish the clinical and epidemiological importance of *C. brasiliensis* in feline medicine.

## Conclusion

The present study documents the occurrence of *C. brasiliensis* in domestic cats in the state of Rio de Janeiro and reinforces that this parasite is not restricted to wild felids in Brazil. Molecular and phylogenetic analyses support the identification of the agent, particularly when considering the *cytB* gene. Although the positive cats showed relevant hematological alterations, the results should be interpreted with caution, given the small number of cases and the limitations inherent to the retrospective study design. Nevertheless, this study expands the current knowledge of the diversity of *Cytauxzoon* in Brazilian felids and highlights the need for further investigations into pathogenicity, vector competence, and the interface between domestic and wild hosts.

## Supplementary Information

Below is the link to the electronic supplementary material.

Supplementary Material 1

## Data Availability

The data supporting the findings of this study are available from the corresponding author upon reasonable request.
