# Supplementary material for: Molecular characterization of Cytauxzoon brasiliensis in domestic cats (Felis catus) from Rio de Janeiro state, Brazil: hematological findings
Source: Vet Res Commun. 2026 Jul 7;50(5):443. doi: 10.1007/s11259-026-11385-z (PMC13342325; doi:10.1007/s11259-026-11385-z)
Supplement: Supplementary file 1 — Supplementary Material 1 [file 11259_2026_11385_MOESM1_ESM.pdf]

Supplementary Material 1

Molecular detection of piroplasmids based on the 18S rRNA gene

Samples were subjected to nested-PCR assays for amplification of an approximately 800 bp fragment of the 18S rRNA gene. In the first reaction, 3 µL of extracted DNA was used in a mixture containing 1X buffer (Colorless GoTaq®, Promega®), 1.5 mM $MgCl_2$ (Promega® $MgCl_2$ Solution), 0.2 mM dNTPs, 0.8 µM of each primer BTF1 (5' GGCTCATTACAACAGTTATAG 3') and BTR1 (5' CCCAAAGACTTTGATTTCTCTC 3'), and 1U of Taq DNA polymerase (GoTaq®, Promega®) per reaction. Thermocycling conditions consisted of an initial denaturation at 95 °C for 2 minutes and 30 seconds, followed by 30 cycles of 94 °C for 30 seconds, 58 °C for 20 seconds, and 72 °C for 30 seconds, with a final extension at 72 °C for 5 minutes.

In the second reaction, 2 µL of the first PCR product was used as template, maintaining the same final reagent concentrations: 1X buffer (Colorless GoTaq®, Promega®), 1.5 mM $MgCl_2$ (Promega® $MgCl_2$ Solution), 0.2 mM dNTPs, 0.8 µM of each primer BTF2 (5' CCGTGCTAATTGTAGGGCTAATAC 3') and BTR2 (5' GGACTACGACGGTATCTGATCG 3'), and 1U of Taq DNA polymerase (GoTaq®, Promega®) per reaction. Thermocycling conditions consisted of an initial denaturation at 95 °C for 2 minutes and 30 seconds, followed by 30 cycles of 94 °C for 30 seconds, 62 °C for 20 seconds, and 72 °C for 30 seconds, with a final extension at 72 °C for 5 minutes.

Molecular characterization of *Cytauxzoon* spp. based on the *cytB* gene

Samples were subjected to nested-PCR assays using the external primers CytbF1 (5'-CTTAACCCAACTCACGTACC-3') and CytbR3 (5'-GGTTAATCTTTCCTATTCCTTACG-3'), and the internal primers CytbFinn (5'-ACCTACTAAACCTTATTCAAGCRTT-3') and CytbRinn (5'-AGACTCTTAGATGYAAACTTCCC-3'), for amplification of an approximately 1333 base pair (bp) fragment of the cytB gene, in a final reaction volume of 25 µL.

In the first reaction, 3 µL of extracted DNA was used in a mixture containing 1X buffer (Colorless GoTaq®, Promega®), 2.5 mM $MgCl_2$ (Promega® $MgCl_2$ Solution), 0.2 mM dNTPs, 0.8 µM of each primer, and 1 U of Taq DNA polymerase (GoTaq®, Promega®). Thermocycling conditions consisted of an initial denaturation at 95 °C for 5 minutes, followed by 35 cycles of 95 °C for 30 seconds, 53 °C for 45 seconds, and 72 °C for 60 seconds, with a final extension at 72 °C for 7 minutes.

In the second reaction, 2 µL of the first PCR product was used as template, maintaining the same final reagent concentrations: 1X buffer (Colorless GoTaq®, Promega®), 2.5 mM $MgCl_2$ (Promega® $MgCl_2$ Solution), 0.2 mM dNTPs, 0.8 µM of each primer, and 1 U of Taq DNA polymerase (GoTaq®, Promega®). Thermocycling conditions consisted of an initial denaturation at 95 °C for 5 minutes, followed by 35 cycles of 95 °C for 30 seconds, 55 °C for 45 seconds, and 72 °C for 60 seconds, with a final extension at 72 °C for 7 minutes.

Molecular detection of *Bartonella* spp. based on the ITS gene

Samples were subjected to conventional PCR assays using the primers QVE1 (5'-TTCAGATGATGATCCCAAGC-3') and QVE3 (5'-AACATGTCTGAATATATCTTC-3') (Renesto et

al. 2001), for amplification of an approximately 400–600 base pair (bp) fragment of the ITS gene, in a final reaction volume of 25 µL.

The reactions were performed using 2 µL of extracted DNA in a mixture containing 1X buffer (Colorless GoTaq®, Promega®), 2.0 mM $MgCl_2$ (Promega® $MgCl_2$ Solution), 0.2 mM dNTPs, 0.8 µM of each primer, and 1 U of Taq DNA polymerase (GoTaq®, Promega®). Thermocycling conditions consisted of an initial denaturation at 94 °C for 5 minutes, followed by 40 cycles of 94 °C for 20 seconds, 52 °C for 20 seconds, and 72 °C for 30 seconds, with a final extension at 72 °C for 5 minutes.

Molecular detection of members of the class Mollicutes based on the 16S rRNA gene

Conventional PCR was performed using the primers MGSO (5’-TGCACCATCTGTCACTCTGTTAACCTC-3’) and GPO3 (5’-GGGAGCAAACAGGATTAGATACCCT-3’) (van Kuppeveld et al. 1994), for amplification of an approximately 270 base pair (bp) fragment of the 16S rRNA gene, in a final reaction volume of 25 µL.

The reactions were performed using 1X buffer (Colorless GoTaq®, Promega®), 2.0 mM $MgCl_2$ (Promega® $MgCl_2$ Solution), 0.2 mM dNTPs, 0.4 µM of each primer, and 1 U of Taq DNA polymerase (GoTaq®, Promega®). Thermocycling conditions consisted of an initial denaturation at 94 °C for 5 minutes, followed by 40 cycles of 94 °C for 30 seconds, 55 °C for 30 seconds, and 72 °C for 30 seconds, with a final extension at 72 °C for 5 minutes.

Molecular detection of agents of the family Anaplasmataceae based on the 16S rRNA gene

Conventional PCR was performed using the primers EHR16SD (5’-GGTACCYACAGAAGAAGTCC-3’) and EHR16SR (5’-TAGCACTCATCGTTTACAGC-3’) (Parola et al., 2000), for amplification of an approximately 345 base pair (bp) fragment of the 16S rRNA gene, in a final reaction volume of 25 µL.

The reactions were performed using 1X buffer (Colorless GoTaq®, Promega®), 1.5 mM $MgCl_2$ (Promega® $MgCl_2$ Solution), 0.2 mM dNTPs, 0.6 µM of each primer, and 1 U of Taq DNA polymerase (GoTaq®, Promega®). Thermocycling conditions consisted of an initial denaturation at 95 °C for 5 minutes, followed by 40 cycles of 95 °C for 15 seconds, 55 °C for 15 seconds, and 72 °C for 30 seconds, with a final extension at 72 °C for 5 minutes.

Molecular detection of *Leishmania infantum chagasi* based on the 18S rRNA gene

Samples were subjected to nested-PCR assays using the primers R221 (5’-GGTTCCTTTCCTGATTTACG-3’) and R332 (5’-GGCCGGTAAAGGCCGAATAG-3’), for amplification of an approximately 603 base pair (bp) fragment of the 18S rRNA gene (Lignon et al. 2024), in a final reaction volume of 25 µL.

The reactions were performed using 1X buffer (Colorless GoTaq®, Promega®), 2.5 mM $MgCl_2$ (Promega® $MgCl_2$ Solution), 0.2 mM dNTPs, 0.8 µM of each primer, and 1 U of Taq DNA polymerase (GoTaq®, Promega®). Thermocycling conditions consisted of an initial denaturation at 94 °C for 2 minutes, followed by 35 cycles of 94 °C for 30 seconds, 55 °C for 30 seconds, and 72 °C for 30 seconds, with a final extension at 72 °C for 10 minutes.

For the second reaction, the primers R222 (5'-TATTGGAGATTATGGAGCTG-3') and R333 (5'-AAAGCGGGCGCGGTGCTG-3') were used to amplify an approximately 358 bp fragment, maintaining a final reaction volume of 25 µL. The reactions contained 1X buffer (Colorless GoTaq®, Promega®), 2.5 mM $MgCl_2$, 0.2 mM dNTPs, 0.8 µM of each primer, and 1 U of Taq DNA polymerase (GoTaq®, Promega®).

Thermocycling conditions consisted of an initial denaturation at 94 °C for 2 minutes, followed by 35 cycles of 94 °C for 30 seconds, 60 °C for 30 seconds, and 72 °C for 30 seconds, with a final extension at 72 °C for 10 minutes.

References

Lignon JS, Pinto DM, Teles MA, Trindade MAC, Portela PR, Monteiro SG, Martins KR, Cunha RC, Pappen FG, Bohm BC, Bruhn FRP 2024 Absence of *Leishmania* spp. DNA in road-killed wild mammals in Southern Brazil. Rev. Bras. Parasitol. Vet. 33: e007924. doi: 10.1590/S1984-29612024038

Parola P, Roux V, Camicas JL, Brouqui P, Raoult D 2000 Detection of ehrlichiae in African ticks by polymerase chain reaction. Trans. R. Soc. Trop. Med. Hyg. 94: 707–708. doi: 10.1016/S0035-9203(00)90243-8

Renesto P, Gouvernet J, Drancourt M, Roux V, Raoult D 2001 Use of *rpoB* gene analysis for detection and identification of *Bartonella* species. J. Clin. Microbiol. 39: 430–437. doi: 10.1128/JCM.39.2.430-437.2001

van Kuppeveld FJM, Johansson KE, Galama JMD, Kissing J, Bolske G, van der Logt JTM, Melchers WJG 1994 Detection of *mycoplasma* contamination in cell cultures by a *mycoplasma* group-specific PCR. Appl. Environ. Microbiol. 60: 149–152. doi: 10.1128/AEM.60.1.149-152.1994

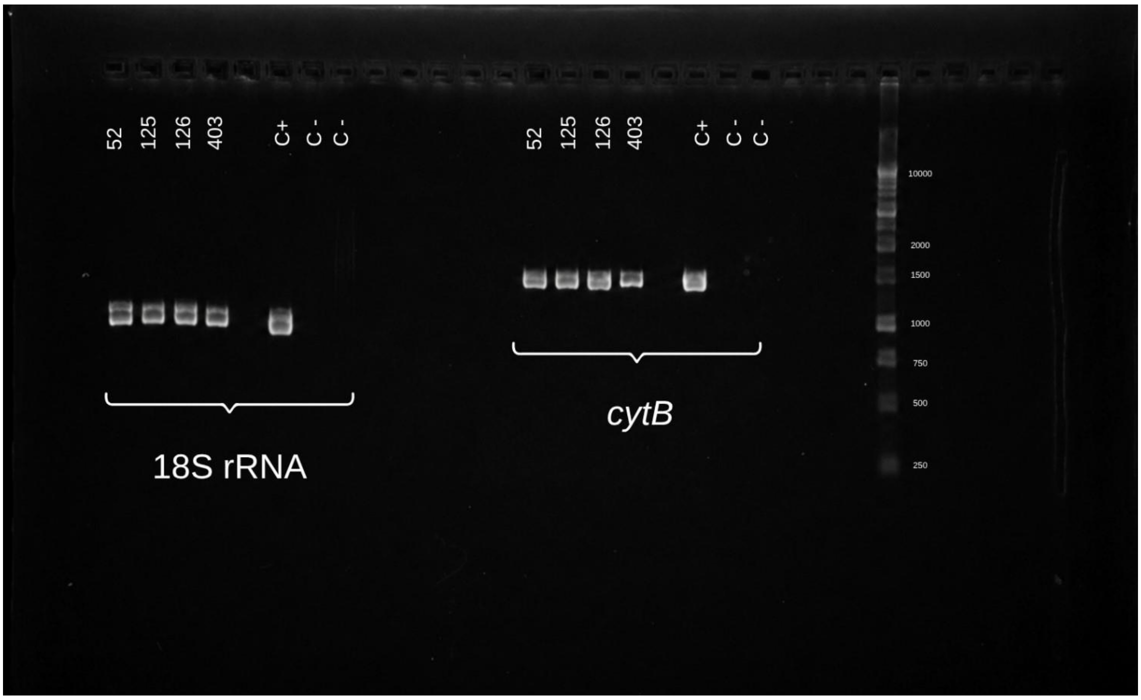

Figure S1. Agarose gel electrophoresis of PCR products obtained from the amplification of *Cytauxzoon brasiliensis* DNA. (A) 18S rRNA gene (~800 bp). (B) *cytB* gene (~1,444 bp). Sample identities are indicated directly above the corresponding wells. The gels include four positive samples, one positive control, two negative controls, and a 1 kb DNA Ladder (Promega).
